# Infection of ferrets with wild type-based recombinant canine distemper virus overwhelms the immune system and causes fatal systemic disease

**DOI:** 10.1128/msphere.00082-23

**Published:** 2023-06-28

**Authors:** Brigitta M. Laksono, Dagmar Roelofs, Anouskha D. Comvalius, Katharina S. Schmitz, Laurine C. Rijsbergen, Daryl Geers, Sham Nambulli, Peter van Run, W. Paul Duprex, Judith M. A. van den Brand, Rory D. de Vries, Rik L. de Swart

**Affiliations:** 1 Department of Viroscience, Erasmus MC, Rotterdam, the Netherlands; 2 Department of Biomolecular Health Sciences, Division of Pathology, Universiteit Utrecht, Utrecht, the Netherlands; 3 Centre for Vaccine Research, University of Pittsburgh School of Medicine, Pittsburgh, Pennsylvania, USA; 4 Department of Virology, Wageningen Bioveterinary Research, Lelystad, the Netherlands; University of Kentucky College of Medicine, Lexington, Kentucky, USA

**Keywords:** morbillivirus, canine distemper virus, immunopathogenesis, ferrets

## Abstract

Canine distemper virus (CDV) causes systemic infection resulting in severe and often fatal disease in a large spectrum of animal host species. The virus is closely related to measles virus and targets myeloid, lymphoid, and epithelial cells, but CDV is more virulent and the infection spreads more rapidly within the infected host. Here, we aimed to study the pathogenesis of wild-type CDV infection by experimentally inoculating ferrets with recombinant CDV (rCDV) based on an isolate directly obtained from a naturally infected raccoon. The recombinant virus was engineered to express a fluorescent reporter protein, facilitating assessment of viral tropism and virulence. In ferrets, this wild type-based rCDV infected myeloid, lymphoid, and epithelial cells, and the infection resulted in systemic dissemination to multiple tissues and organs, especially those of the lymphatic system. High infection percentages in immune cells resulted in depletion of these cells both from circulation and from lymphoid tissues. The majority of CDV-infected ferrets reached their humane endpoints within 20 d and had to be euthanized. In that period, the virus also reached the central nervous system in several ferrets, but we did not observe the development of neurological complications during the study period of 23 d. Two out of 14 ferrets survived CDV infection and developed neutralizing antibodies. We show for the first time the pathogenesis of a non-adapted wild type-based rCDV in ferrets.

**IMPORTANCE:**

Infection of ferrets with recombinant canine distemper virus (rCDV) expressing a fluorescent reporter protein has been used as proxy to understand measles pathogenesis and immune suppression in humans. CDV and measles virus use the same cellular receptors, but CDV is more virulent, and infection is often associated with neurological complications. rCDV strains in current use have complicated passage histories, which may have affected their pathogenesis. Here, we studied the pathogenesis of the first wild type-based rCDV in ferrets. We used macroscopic fluorescence to identify infected cells and tissues; multicolor flow cytometry to determine viral tropism in immune cells; and histopathology and immunohistochemistry to characterize infected cells and lesions in tissues. We conclude that CDV often overwhelmed the immune system, resulting in viral dissemination to multiple tissues in the absence of a detectable neutralizing antibody response. This virus is a promising tool to study the pathogenesis of morbillivirus infections.

## INTRODUCTION

Canine distemper virus (CDV) is a member of the genus *Morbillivirus*, family *Paramyxoviridae*, and causes severe disease in a broad range of host species (1 - 4). The virus uses the cellular receptors CD150 (also known as signaling lymphocyte activating molecule family member 1 or SLAMF1) and nectin-4 to infect immune cells and epithelial cells, respectively. Infected animals show a range of clinical signs, such as fever, nasal and ocular discharge, conjunctivitis, and pneumonia. Rapid and widespread infection of immune cells results in severe immune suppression, leading to increased risk of secondary infections. CDV infection often results in neurological complications and death (5).

Morbillivirus infections in susceptible, immunologically naive, domesticated, and wild animals often lead to high morbidity and mortality. The eradicated rinderpest virus, which infected ungulates, had a mortality rate up to 100% in cattle. *Peste des petits ruminants* virus, phocine distemper virus (PDV), and cetacean morbillivirus (CeMV) cause severe disease with high mortality rates in small ruminants, seals, and cetaceans, respectively. Interspecies transmission has also been reported: CDV is often transmitted between various host species, and CeMV was described to be transmitted from a dolphin to a seal (6). Measles virus (MV) is also a morbillivirus, which causes disease in humans and non-human primates (NHPs), but the frequency of fatal disease is lower than that associated with animal morbilliviruses. While CDV-infected hosts experience almost complete depletion of lymphocytes during the peak of viremia and succumb to the disease before any immune responses are detected, MV-infected NHPs or humans mostly develop a self-limiting infection with milder viremia, and strong and protective MV-specific immune responses (7).

CDV pathogenesis studies in ferrets using recombinant viruses expressing fluorescent reporter proteins have mostly been performed with three virus strains. The highly neurotropic Snyder-Hill (rCDV^SH^) strain, which was passaged several times in dog brains, is known to rapidly induce fatal disease associated with meningoencephalitis in ferrets (8). The rCDV^5804P^ strain is adapted to ferrets and, although not as virulent as CDV^SH^, still leads to fatal disease and focal brain infection in ferrets (9). The rCDV^R252^ strain, based on an isolate from a dog with demyelinating encephalitis, induced slower disease progression and lower levels of lymphopenia in ferrets compared to recombinant viruses based on the CDV^SH^ or CDV^5804P^ strain (8). The expression of a fluorescent reporter protein was previously shown to have no influence on virulence when introduced as an additional transcription unit (ATU) in the sixth position of the genome (10). rCDV^SH^, for example, has been rescued expressing enhanced green fluorescent protein, Venus, dTomato, or blue fluorescent protein, and was used to assess systemic viral spread and transmission *in vivo* (8, 11). In these *in vivo* models, CDV-infected tissues and organs were identified macroscopically by detection of fluorescence, and the number of infected cells was quantified using flow cytometry.

Recently, recombinant CDV (rCDV) based on the consensus sequence of an early passage virus isolate from a naturally infected raccoon in Rhode Island (USA) was generated (rCDV^RI^) (12). This virus has a minimal *in vitro* passage history in CD150^+^ cells. Here, we inoculated ferrets (*Mustela putorius furo*) with rCDV^RI^ engineered to express a fluorescent reporter protein from an ATU inserted in position 6 of the viral genome [rCDV^RI^Venus(6)], and assessed the pathogenesis of canine distemper in these animals.

## MATERIALS AND METHODS

### Viruses and cells

The rCDV expressing Venus fluorescent reporter protein from an ATU at position 6 of the genome was based on CDV strain Rhode Island isolated from a naturally infected raccoon (12). The stock was grown on Vero-dogCD150 cells (a kind gift from Dr. Y. Yanagi, Kyushu University, Fukuoka, Japan) (13) or on canine lymphoblastoid cell line (CLBL) (14). Vero-dogCD150 cells were grown in Dulbecco’s modified Eagle medium, and CLBL were grown in RPMI-1640. All culture media were supplemented with 10% fetal bovine serum, penicillin (100 IU/mL), streptomycin (100 µg/mL), and glutamine (2 mM). Animals in the control group were either inoculated with recombinant MV strain Khartoum-Sudan expressing Venus from an ATU at position 3 of the genome, which did not result in detectable MV replication or development of MV-specific immune responses, or were uninfected control animals of other studies.

### Animal study design

White blood cells (WBC) and tissues were collected from ferrets (*Mustela putorius furo*) after inoculation with rCDV^RI^Venus(6) (*n* = 20) or from mock control animals (*n* = 9). Male ferrets were exclusively used in this study because of their bigger body size, which allowed a more frequent blood sampling. Animals were intratracheally inoculated with a low dose (1 × 10^4^ TCID_50_) of rCDV^RI^Venus (6). The course of infection was monitored up to 23 days post-inoculation (dpi). Ferrets were euthanized at 2, 4, 6, 8, 10, 13, 14, 15, 16, 17, 20, and 23 dpi.

### Necropsy

Animals were euthanized by exsanguination under ketamine-medetomidine anesthesia. For the purpose of detecting Venus fluorescence macroscopically, a custom-made lamp containing six 5-Volt LEDs (Luxeon Lumileds [Amsterdam, the Netherlands], Lambertian, cyan, peak emission 490–495 nm) mounted with D480/40 bandpass filters (Chroma, Olchin Germany) was used as previously described (15). Photographs were made using a Nikon D80 SLR camera mounted with a Dark Reader camera filter (Clare Chemical Research, Dolores, Colorado, US). Lymphoid tissues were collected in buffered formalin for immunohistochemistry (IHC) or in phosphate-buffered saline (PBS) for preparation of single-cell suspensions for flow cytometry.

### Flow cytometry

Frequencies of infected cells and lymphocyte subsets in peripheral blood mononuclear cells and tissues were determined by flow cytometry using a BD FACSCanto II or a FACSLyric (BD Biosciences, San Jose, California, US). WBC were isolated from whole blood following red blood cell lysis. Leukocytes were isolated from different tissues, which were cut into small pieces and processed further using a gentleMACS (Miltenyi Biotec, Bergisch Gladbach, Germany). Single cell suspensions from the tissues were processed through a 100 µm filter then isolated in PBS. For determination of leukocyte phenotypes, cells were incubated with monoclonal antibodies directed to CD4-APC, CD8-PE-Cy7, CD79a-PerCP, CD11b-AmCyan, and HLA-DR-APC-Cy7, followed by fixation according to the manufacturer’s instructions (BD Biosciences). Unstained cells were fixed with 2% paraformaldehyde prior to flow cytometry measurement. Percentage of infected cells was determined by detection of Venus fluorescent signal. Data were acquired with BD FACSDiva or FACSSuite software and analyzed with FlowJo software.

### Virus neutralization assay

Virus neutralizing antibodies were detected using a fluorescent virus neutralization (VN) assay. Briefly, serial dilutions (2^2^–2^10^, each dilution was tested in duplicate) of heat-inactivated plasma and/or serum samples were incubated with rCDV^RI^Venus(6) (TCID_50_: 60) for 1  h at 37°C. The mixture was added to a preseeded monolayer of Vero-dogCD150 cells and incubated for 7 d at 33°C. The 50% endpoint neutralizing antibody titer was calculated using the Kärber method.

### Histology and IHC

Ferret mandibular and tracheobronchial lymph node (LN), tonsil, trachea, lung, spleen, and brain tissues were fixed by immersion in 10% neutral-buffered formalin, embedded in paraffin, and sectioned at 3 µm. Sequential slides were either stained with hematoxylin and eosin or green fluorescent protein (GFP)^+^ cells were detected by IHC using rabbit polyclonal antibody directed to GFP (Invitrogen, Waltham, Massachussets, US) after antigen retrieval with citrate buffer (10 mM, pH = 6.0) with heat induction. Goat antirabbit antibody conjugated with biotin was used as a secondary antibody and streptavidin-horseradish peroxidase was added for signal detection. The slides were incubated with primary antibody overnight at 4°C before incubation with secondary and tertiary antibodies.

To evaluate the virus tropism throughout the course of rCDV^RI^Venus (6)-infection, we performed a semi-quantitative assessment of the GFP antigen expression. Slides were examined blindly, i.e., without knowledge of the infection allocation of the animals. For every organ, 10 randomly chosen 20× objective fields were examined by light microscopy, with every positive cell type in each organ being scored separately. The percentage of positive cells was estimated per field as: 0: 0%; 1: 1–10%; 2: 11–40%; and 3: >40%. The average of 10 fields was calculated to provide a total rounded-up score per cell type per organ.

To evaluate the histopathologic changes throughout the course of rCDV^RI^Venus(6)-infection, a semi-quantitative assessment was performed of the formalin-fixed paraffin-embedded mandibular and tracheobronchial LN, tonsil, trachea, lung, spleen, and brain tissues. We scored the lesions according to the following protocol: For lymphodepletion, we defined the scores as: 1: none; 2: mild (1–20% of cells depleted); 3: moderate (20–50% of cells depleted); and 4: severe (>50% of cells gone). For necrotic lesions, we subjectively scored: 0: none; 1: mild; 2: moderate; and 3: severe. These lesions were morphologically consistent with necrosis, showed karyolysis and karyorrhexis, loss of cellular structure, and the presence cellular debris. For the severity of inflammation, we scored: 0: no inflammatory cells; 1: low numbers of inflammatory cells; 2: moderate numbers of inflammatory cells; and 3: high numbers of inflammatory cells. The inflammatory cell types included in the scoring are neutrophils, macrophages, lymphocytes, plasma cells, and eosinophils. The anatomical location of inflammation was noted to assess whether the presence of inflammation was associated with a lesion. Inclusion bodies were defined to be about 2–4 µm in size, oval to round in shape, bright eosin red in color, and located in the cytoplasm or nuclei of lymphoid or epithelial cells. Cytoplasmic and/or intranuclear inclusion bodies were scored as: 0: none; 1: 1–10% of cells; 2: 10–40% of cells; and 3: >40% of cells. Slides were examined blindly, i.e., without knowledge of the infection allocation of the animals. For every slide, 10 randomly chosen 40× objective fields were scored. An objective of 40× was chosen to accurately assess the presence of inclusion bodies. For each parameter, the average of rounded-up score of the 10 fields was presented as the final score in the observed organ.

### Statistical analyses

Differences in body temperature, weight loss, and lymphocyte count among CDV-infected ferrets, ferrets that survived infection, and control ferrets were assessed using a mixed-effects model repeated measures analysis. *P*-values for group differences are shown in the figure legends. Differences in viral load in nose, throat, and blood were assessed by calculating the Area Under the Curve (AUC) for each animal, followed by a Mann-Whitney test to determine differences between the groups. All statistical analyses were performed using Graphpad Prism 9.

## RESULTS

### Clinical parameters

Our study included ferrets inoculated intratracheally with rCDV^RI^Venus(6) (*n* = 20) and a mock-inoculated control group (*n* = 9). The course of infection was monitored up to 23 dpi, with several animals euthanized at earlier time points to assess CDV tropism and tissue distribution. Details on animals included in this study are summarized in Table 1.

**TABLE 1:** Animals included in this study. Animals euthanized at 2–10 days post-inoculation (dpi) were sacrificed per protocol to study the pathogenesis of CDV infection at early time points

Group	Animal ID	Virus	dpi	Cause of death	Initial body weight (g)
rCDV^RI^Venus(6) (*n* = 20)	4366	rCDV^RI^Venus(6)	2	Euthanized at early time point	1,177
	1085	rCDV^RI^Venus(6)	4	Euthanized at early time point	1,332
	44407	rCDV^RI^Venus(6)	4	Euthanized at early time point	917
	1675	rCDV^RI^Venus(6)	6	Euthanized at early time point	1,283
	2427	rCDV^RI^Venus(6)	6	Euthanized at early time point	1,308
	40005	rCDV^RI^Venus(6)	6	Euthanized at early time point	1,086
	7392	rCDV^RI^Venus(6)	8	Euthanized at early time point	1,526
	44162	rCDV^RI^Venus(6)	8	Euthanized at early time point	1,228
	4219	rCDV^RI^Venus(6)	10	Euthanized at early time point	1,634
	2683	rCDV^RI^Venus(6)	13	Distemper, euthanized upon reaching humane end point	2,093
	02406	rCDV^RI^Venus(6)	13	Distemper, euthanized upon reaching humane end point	1,092
	8754	rCDV^RI^Venus(6)	14	Distemper, euthanized upon reaching humane end point	1,411
	76232	rCDV^RI^Venus(6)	15	Distemper, euthanized upon reaching humane end point	1,405
	44792	rCDV^RI^Venus(6)	15	Distemper, euthanized upon reaching humane end point	1,222
	5591	rCDV^RI^Venus(6)	16	Distemper, euthanized upon reaching humane end point	1,309
	4041	rCDV^RI^Venus(6)	17	Distemper, euthanized upon reaching humane end point	1,487
	53074	rCDV^RI^Venus(6)	17	Distemper, euthanized upon reaching humane end point	1,592
	53636	rCDV^RI^Venus(6)	20	Distemper, euthanized upon reaching humane end point	1,418
	2671	rCDV^RI^Venus(6)	23	Distemper, survived despite infection, euthanized at the end of the study period	1,414
	1041	rCDV^RI^Venus(6)	23	Distemper, survived despite infection, euthanized at the end of the study period	1,728
Control (*n* = 9)	2725	rMV^KS^Venus(3)	2	Euthanized at early time point	1,624
	8606	rMV^KS^Venus(3)	4	Euthanized at early time point	1,511
	0303	rMV^KS^Venus(3)	6	Euthanized at early time point	1,496
	0560	rMV^KS^Venus(3)	23	Euthanized at the end of the study period	1,438
	1136	rMV^KS^Venus(3)	23	Euthanized at the end of the study period	1,366
	8277	rMV^KS^Venus(3)	23	Euthanized at the end of the study period	1,187
	6200	Uninfected	N/A	Uninfected animal	1,415
	6548	Uninfected	N/A	Uninfected animal	1,808
	51636	Uninfected	N/A	Uninfected animal	1,186

**Fig 1:**
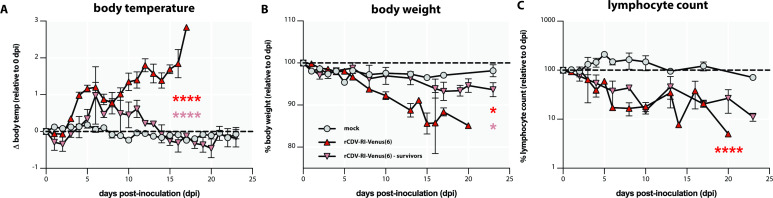
Clinical parameters of ferrets inoculated with rCDV^RI^Venus(6). (A) All canine distemper virus (CDV)-inoculated animals developed significantly higher fever than those of the mock control group (red asterisks). Two ferrets experienced transient fever, which was subdued within a week after its peak. These two ferrets developed significantly lower grade of fever as compared to the CDV-infected group (pink asterisks). (B) CDV-inoculated ferrets lost up to 20% of their body weights, which was significantly different from the control group (red asterisk). In contrast, surviving ferrets only lost around 5% of their body weights. (C) All CDV-inoculated ferrets experienced lymphopenia (significant difference with the mock control group) that lasted throughout the period of the study (red asterisks). Two ferrets inoculated with rCDV^RI^Venus(6) survived the infection and were sacrificed at the end of the study period. *P < 0.1; ****P < 0.0001. Body temp, body temperature.

Clinical progression of canine distemper was monitored through measurement of body temperature and weight. CDV-inoculated ferrets experienced biphasic fever accompanied by a moderate weight loss over time (Fig. 1A and B). No increase of body temperature or substantial loss of body weight was observed in the control group. Some of the animals in our study (9 out of 20 animals) were euthanized at early time points per protocol, to characterize the frequency and phenotype of infected cells (see Table 1). Nine out of the remaining 11 animals inoculated with rCDV^RI^Venus(6) were euthanized upon reaching pre-defined humane endpoints (Table 1), resulting in a case-fatality rate of 82%. The other two animals were able to clear the infection and were euthanized 23 dpi, upon completion of the study. These two animals still developed fever, but their temperature returned to normal and resembled that of the control group from around 12 dpi onward (Fig. 1A). Weight loss in these two ferrets was minimal (Fig. 1B). CDV is known to cause lymphopenia. Ferrets inoculated with CDV rapidly lost a fraction of peripheral blood lymphocytes (Fig. 1C). The percentage of circulating lymphocytes in these animals remained low throughout the course of the infection. The two surviving animals also developed lymphopenia and did not recover their lymphocyte counts within the timeframe of the study. In the control group, the percentages of lymphocytes fluctuated, with a two-fold increase during the first 2 wk post-mock inoculation, with large error margins, followed by a decrease to a level similar to that of the day of inoculation.

### Pathogenesis in the respiratory tract

**Fig 2:**
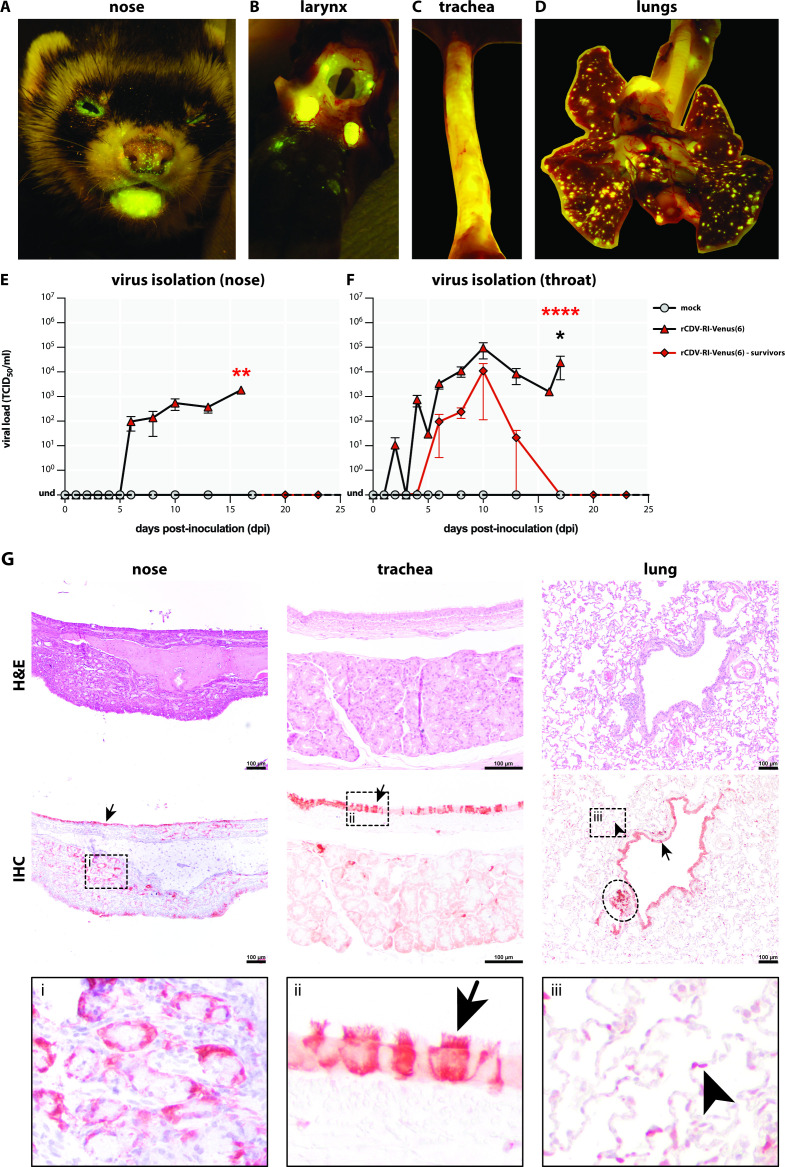
Replication of rCDV^RI^Venus(6) in ferret respiratory tract. (A–D) Focal sites of infection could be detected macroscopically in the upper and lower respiratory tracts. (E and F) Infectious virus was isolated from swabs of the nose and the throat of canine distemper virus-infected ferrets (asterisks indicate statistical comparison with mock control group), but not from control animals. Two ferrets inoculated with rCDV^RI^Venus(6) were able to clear the infection from the respiratory tract (black asterisk indicates comparison with mock control group). (G) Epithelial cells of the upper and lower respiratory tracts were infected with rCDV^RI^Venus(6) (arrow), with the highest number of infected cells were found in the trachea and bronchi of the lung. Infected cells were also present in (i) seromucous glands in the nose (representative figure from 17 dpi), (ii) ciliated epithelial cells of the trachea (representative figure from 4 dpi), and bronchus-associated lymphoid tissues (ellipse), and (iii) pneumocytes in the lungs (representative figure from 6 dpi). Scale bar: 100 µm. *P < 0.1; **P < 0.05; ****P < 0.0001. dpi, days post-inoculation; H&E, hematoxylin and eosin staining; IHC, immunohistochemistry; Und, undetectable.

The course of CDV infection in the respiratory tract was monitored by detection of fluorescence. Focal sites of infection were observed macroscopically in the nose (Fig. 2A). Tonsils, trachea, and lungs were also positive (Fig. 2B through D). No fluorescence was detected in the animals of the control group. Infection resulted in production of infectious virus, which was isolated from nose (Fig. 2E) and throat (Fig. 2F) swabs collected from CDV-inoculated animals on a regular basis. In animals with a fatal infection, infectious virus was isolated until euthanasia, whereas in the two surviving ferrets, viral loads peaked at 10 dpi and decreased steadily to an undetectable level afterward. More infectious virus was isolated from the throat than from the nose in general, and virus was not isolated from the nose of the animals that survived CDV infection.

**Fig 3:**
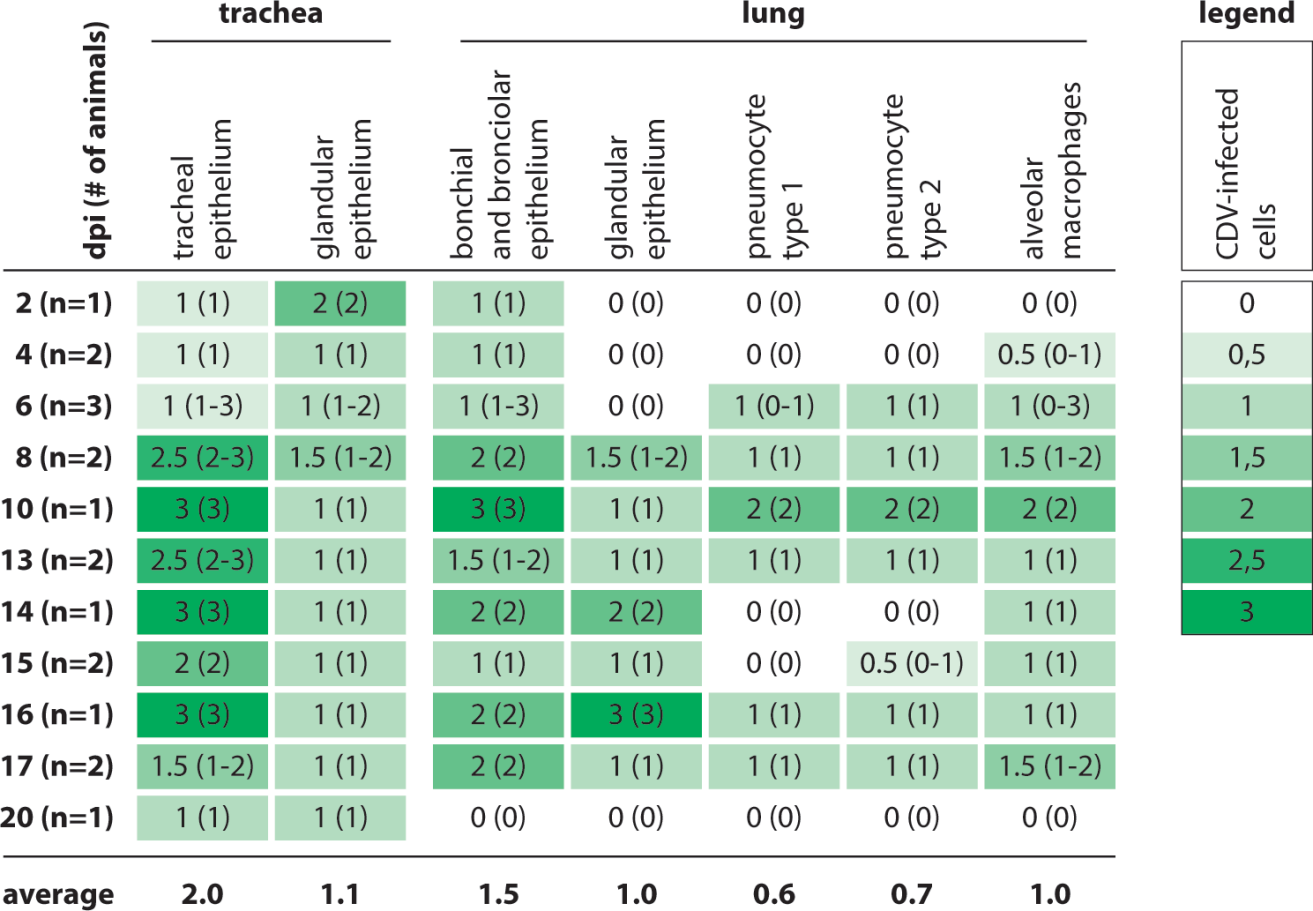
Semi-quantitative scoring of rCDV^RI^Venus(6)-infected cells in the respiratory tract. Canine distemper virus (CDV)-infected cells scores: 0: none; 1: 1–10% CDV^+^ cells; 2: 11–40% CDV^+^ cells; 3: >40% CDV^+^ cells. Median scores with ranges were shown for detection of CDV-infected cells. Cells are color-coded from light (low score) to dark (high score). dpi: days post-inoculation.

**Fig 4:**
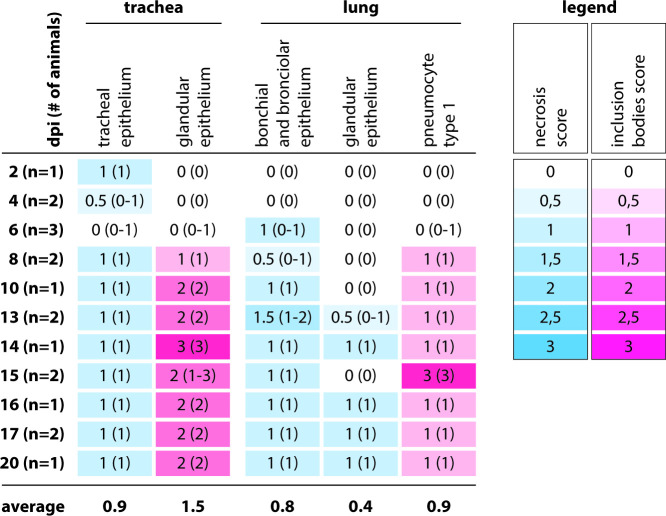
Semi-quantitative scoring of necrosis and number of cells with inclusion bodies in rCDV^RI^Venus(6)-infected ferret respiratory tracts. Necrosis scores: 0: none; 1: mild; 2: moderate; 3: severe. Inclusion body scores: 0: none; 1: 1–10% of cells; 2: 11–40% of cells; 3: >40% of cells. Median scores with ranges were shown for detection of necrosis and inclusion bodies. Cells are color-coded from light (low score) to dark (high score). dpi: days post-inoculation.

To determine the viral tropism and the pathological lesions of CDV infection in the respiratory tract, the trachea and lungs of animals inoculated with rCDV^RI^Venus(6) were semi-quantitatively scored for the number of infected cells, necrosis, and the number of cells with inclusion bodies in histology and IHC (Fig. 3 and 4). Infected cells were observed in low numbers from 2 dpi onward in the trachea and lung, increased in numbers in the respiratory tract epithelia of the animals over the course of time, and remained observable until around 16 dpi (Fig. 3). The majority of CDV-infected cells were epithelial cells in the trachea and bronchi or bronchioles (Fig. 2G). Infected cells were also detected in the nose (ciliated epithelial and seromucous gland cells), trachea (seromucous gland cells), and lungs (pneumocytes and perivascular infiltrating cells) (Fig. 2G i, ii and iii). The number of necrotic cells did not change over time, while the number of cells with inclusion bodies peaked at around 14 or 15 dpi in the ferret trachea and lung, respectively (Fig. 4).

### Systemic CDV infection

**Fig 5:**
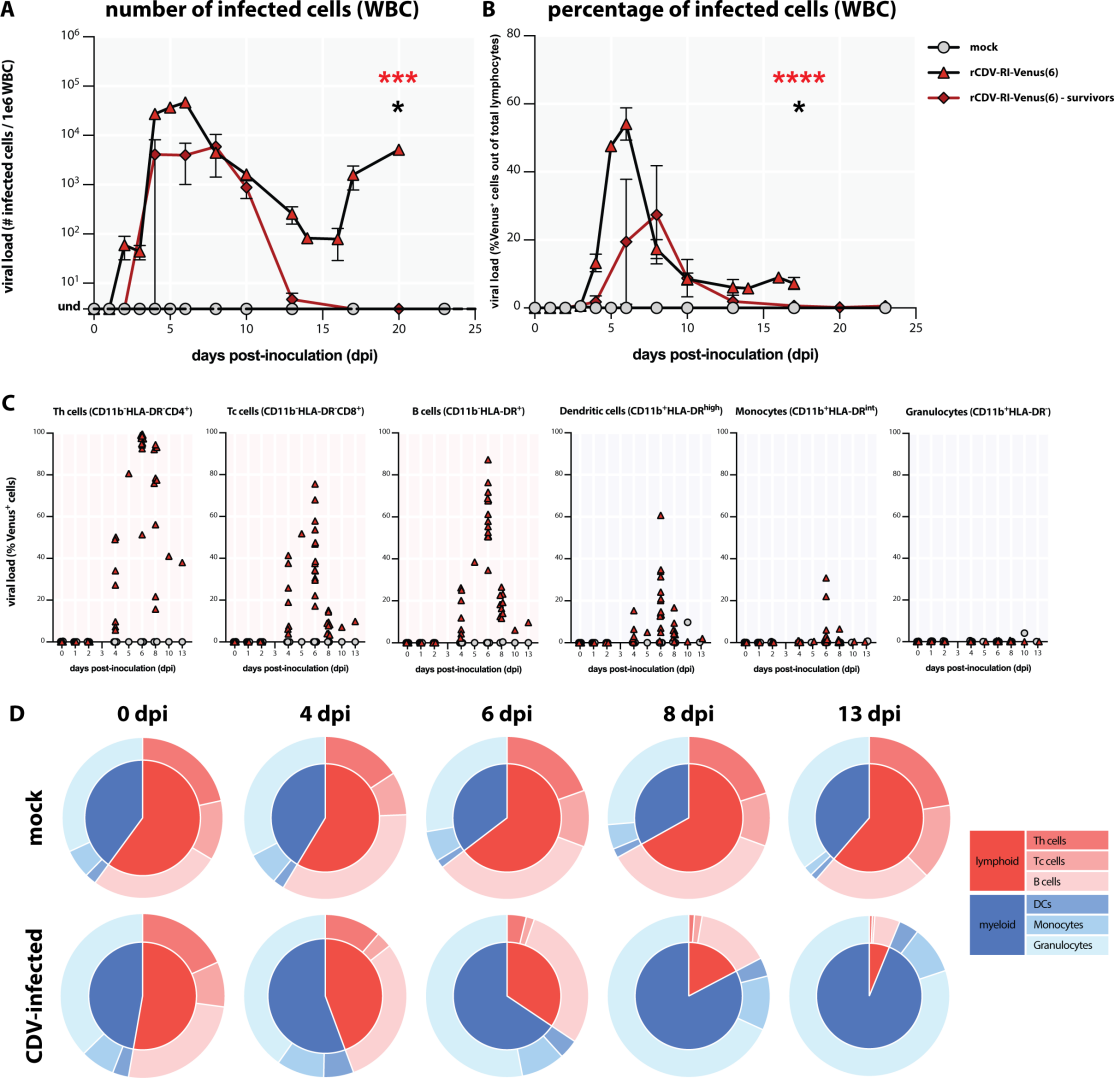
Inoculation with rCDV^RI^Venus(6) resulted in cell-associated viremia and depletion of lymphocytes. (**A**) Number and (**B**) percentages of rCDV^RI^Venus(6)-infected cells in circulating white blood cells (WBC). Canine distemper virus (CDV)-infected cells were initially detected around 3 days post-inoculation (dpi) and reached the peak around 6 dpi (significantly higher than control group; red asterisks). Although rCDV^RI^Venus(6)-infected WBC could be detected and isolated from the two survivor ferrets (significantly higher than control group; black asterisk), the viral loads were lower and became undetectable by 13 dpi. No infected cells were detected in the control animals. (**C**) Identification of the phenotypes of CDV-infected cells in circulating WBC up to 13 dpi. The infected cells were mainly lymphoid cells, with myeloid cells making up a smaller fraction. Individual symbol represents each animal. Triangles, rCDV^RI^Venus(6)-infected animals; circles: control animals. (**D**) Infection of lymphocytes resulted in depletion of CD4^+^ helper T (Th), CD8^+^ cytotoxic T (Tc), and B cells. Pie charts represent relative percentages of lymphoid and myeloid cells in ferret WBC. *P < 0.1; ***P < 0.005; ****P < 0.0001. DCs, dendritic cells.

To assess the level of viremia in CDV-infected animals, we isolated WBC at regular time intervals and determined the number of productively infected cells by virus isolation (Fig. 5A) and the percentage of fluorescent cells by flow cytometry (Fig. 5B). In rCDV^RI^Venus(6)-inoculated animals, we found that the viral load increased sharply and peaked around 6 dpi. The viral load decreased gradually the following days, most probably due to the depletion of target cells, but remained detectable. In the two surviving ferrets, the viral loads peaked lower (10-fold at 6 dpi in virus isolation assays) and later, and CDV became undetectable in WBC in the third week after inoculation.

We then assessed the phenotypes of CDV-infected cells over time by multicolor flow cytometry (Fig. 5C). Infected CD4^+^ helper and CD8^+^ cytotoxic T cells (Th and Tc, respectively) were detected at 4 dpi; infection peaked 2 d later. HLA-DR^+^ B cells were also susceptible to infection and followed similar kinetics. Among peripheral myeloid cells, dendritic cells (DCs, defined in this study as HLA-DR^high^) were found infected but at a lower frequency than lymphoid cells. Interestingly, CD11b^+^ monocytes were clearly less susceptible to infection and only found positive at 6 dpi in few animals. These infected cells rapidly disappeared from the periphery. No CDV-infected peripheral blood granulocytes were detected throughout the study.

**Fig 6:**
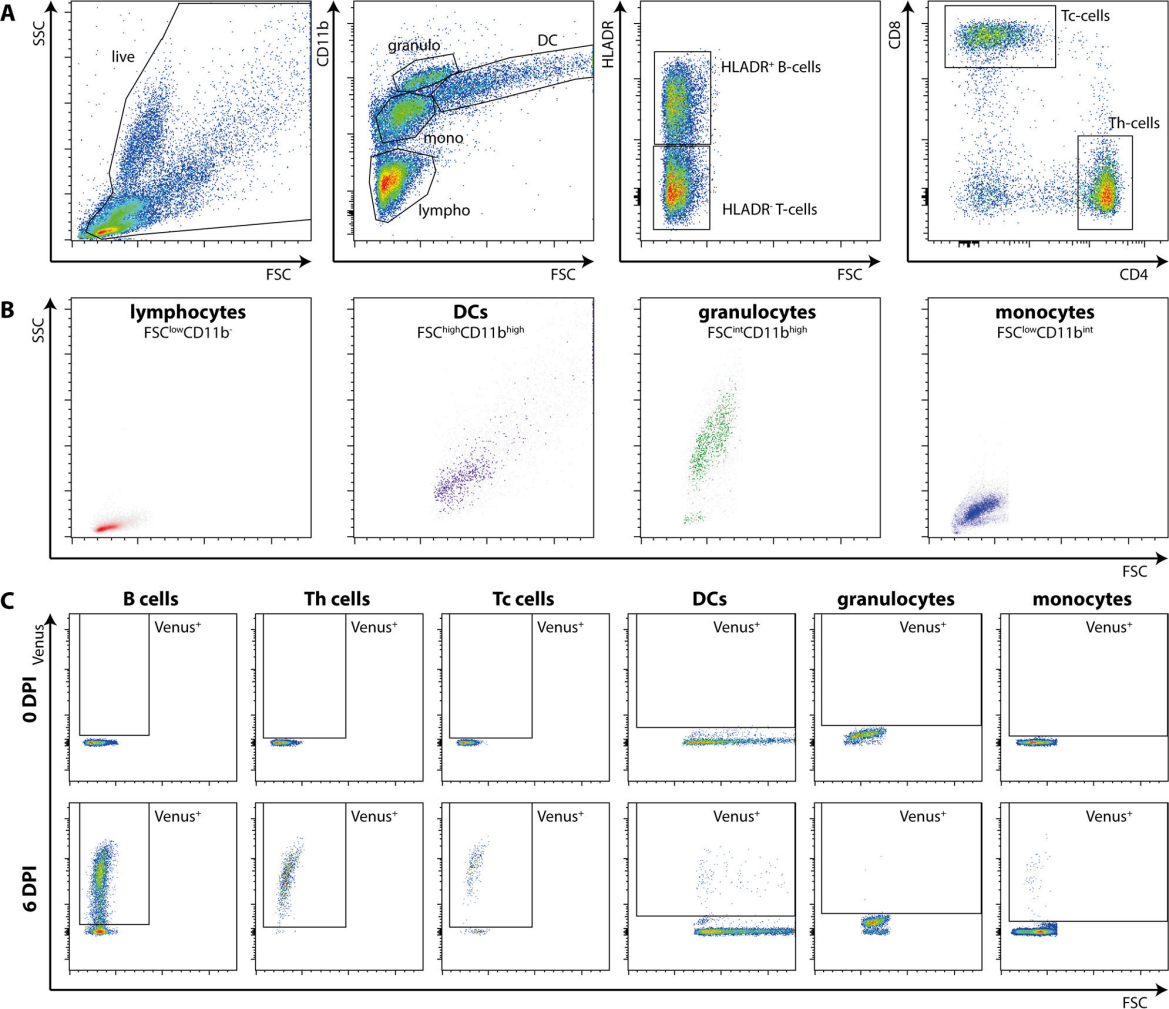
Gating strategies and cell definitions used in flow cytometry. (**A**) Representative gating strategy of ferret peripheral blood mononuclear cells (PBMC). Cells were distinguished based on size (forward scatter, FSC) and granularity (side scatter, SSC), then based on the combination of CD11b and size (FSC). Lymphocytes were further divided into human leukocyte antigen-DR isotype (HLA-DR)^+^ B cells and HLA-DR^-^ T cells. T cells were subdivided into CD4^+^ helper T (Th) and CD8^+^ cytotoxic T (Tc) cells. Lymphocytes were defined as FSC^low^CD11b^-^, dendritic cells (DCs) as FSC^high^CD11b^high^, granulocytes as FSC^int^CD11b^high^, and monocytes as FSC^low^CD11b^int^. Myeloid cells were additionally examined for HLA-DR expression (not shown). (**B**) Back-gating of the representative populations into the FSC-SSC plots demonstrated that the populations have the expected scatter properties. (**C**) Representative gating strategy of Venus^+^ CDV-infected cells in each defined subset at 0 and 6 dpi. dpi, days post-inoculation.

To evaluate the dynamics of CDV-induced viremia and lymphopenia, we assessed the relative size of different immune cell subsets over time (Fig. 5D). In control animals, the relative percentages of lymphoid and myeloid subsets remained stable throughout the study period. In contrast, in CDV-infected animals, the relative percentages of lymphocytes decreased sharply. Combined with the sharp drop in absolute lymphocyte numbers (Fig. 1C), this demonstrates that CDV induced a strong and reproducible depletion of circulating lymphocytes within approximately 1 wk of infection. CDV infection of Th and Tc cells already decreased the population sizes at 4 dpi. Despite the detection of CDV-infected B cells at 4 dpi, the loss of B cells was less pronounced and only detectable at 8 dpi. Gating strategies to define the phenotypes of immune cells in flow cytometry are presented in Fig. 6.

### CDV infection of peripheral tissues and organs

**Fig 7:**
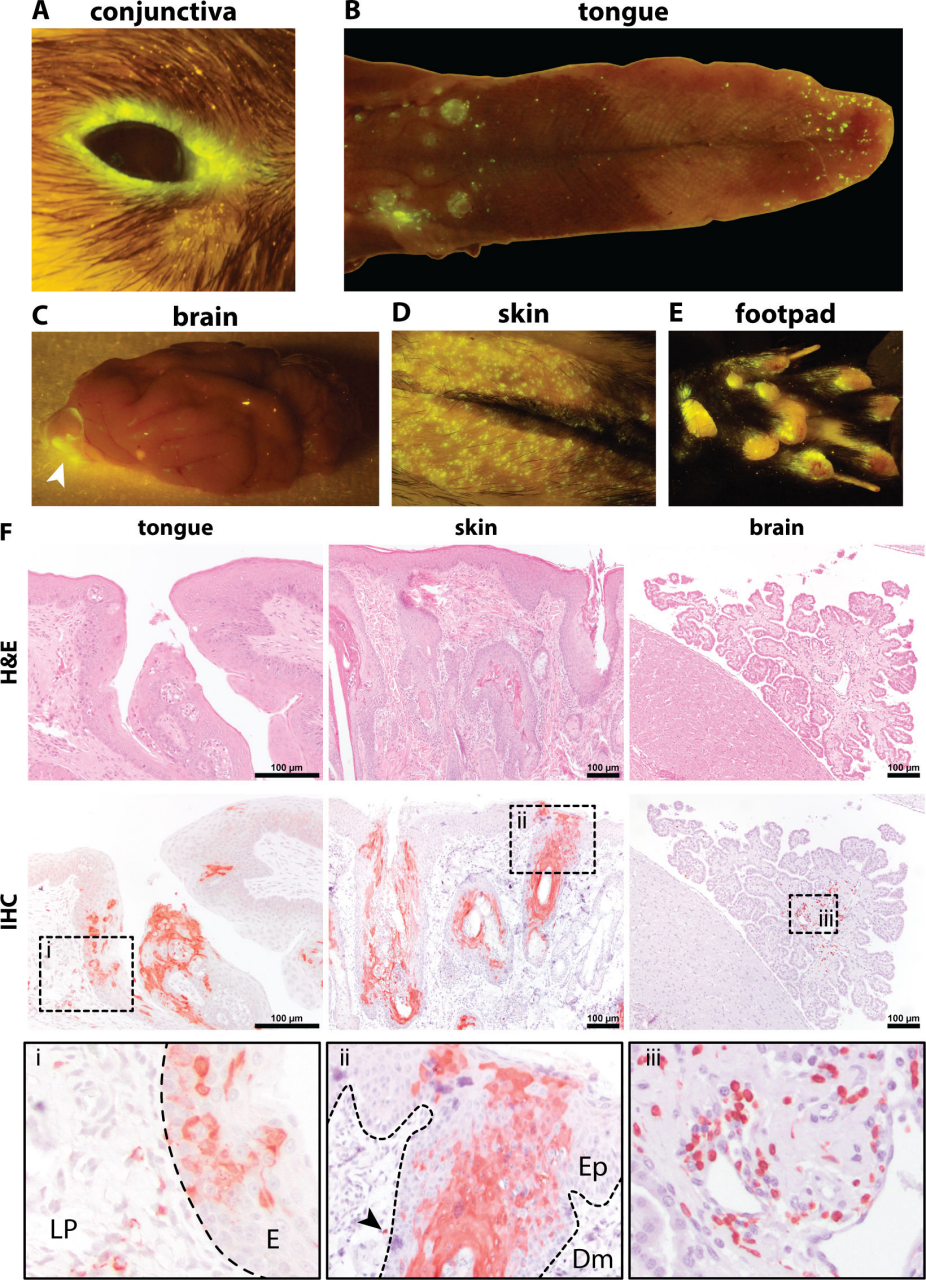
Systemic dissemination of rCDV^RI^Venus(6) led to infection of various peripheral tissues and organs. Focal fluorescent infection sites could be detected in the mucosal sites, such as (**A**) the conjunctiva and (**B**) the tongue at 6 days post-inoculation (dpi). (**C**) In some ferrets, the infection reached the central nervous system at 13 and 15 dpi, although the infection was limited to the olfactory bulb. At 20 dpi, the infection reached the integument, such as (**D**) the skin or (**E**) the footpad. (**F**) Infected cells were detected in the epithelium (i; **E**) and lamina propria (i; LP) of the tongue at 6 dpi; the epidermal (ii; Ep) keratinocytes around the hair follicles and dermal (ii; Dm) cells of the skin (arrow head in inset ii) at 17 dpi. In some ferrets, infected cells were also detected in the choroid plexus of the brain at 15 dpi (iii). Scale bar: 100 µm. H&E, hematoxylin and eosin; IHC, immunohistochemistry.

Viremia led to focal infection of different cells in various peripheral tissues and organs. Fluorescent foci were often observed at mucosal sites, such as the conjunctiva (Fig. 7A) and tongue at 6 dpi (Fig. 7B). We also macroscopically observed infection of the central nervous system (CNS) in two animals, especially in the olfactory bulb at 13 (data not shown) and 15 dpi (Fig. 7C). At the later stage of the CDV infection (20 dpi), epithelial cells and keratinocytes became infected and focal infection was detected in the skin (Fig. 7D) and footpads (Fig. 7E) of the animals. CDV-infected cells were detected by IHC in the tongue (epithelium and lamina propria) and skin (epidermal keratinocytes around the hair follicles and dermal cells) (Fig. 7F). The infected cells in the dermis had the morphology of immune cells. Semi-quantitative measurement of CDV-infected cells in formalin-fixed paraffin-embedded brain tissues showed between 1 and 10% infected cells in the choroid plexus of four out of nine examined ferret brains (Fig. 7F). In three of these, infected cells were also detected in the meninges. To a lesser degree, CDV-infected cells were present in the meningeal blood vessels or the molecular layer of the cerebellum (data not shown). Although infection in the CNS of several animals was detected, no neurological signs were observed within the time frame of this study.

### CDV overwhelms the immune system

**Fig 8:**
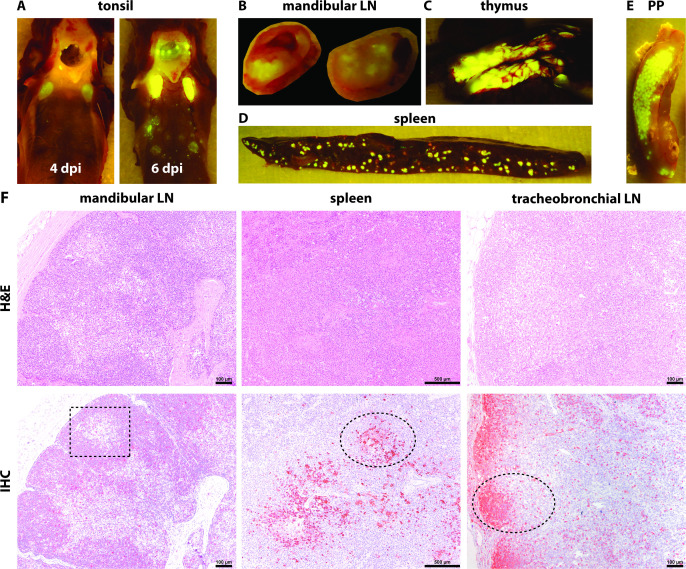
rCDV^RI^Venus(6) spreads throughout the lymphatic system. Fluorescent sites of infection were macroscopically detected not only in lymph nodes (LNs) in close proximity to the respiratory tract, such as (**A**) tonsil and (**B**) mandibular LN, but also more distant, peripheral ones, such as (**C**) thymus, (**D**) spleen, and (**E**) the Peyer’s patches. (**F**) Infected cells (ellipse) and lymphodepletion (rectangle) were observable in the mandibular LN at 6 days post-inoculation (dpi) and spleen at 8 dpi. Follicular structures were hardly recognizable in the tracheobronchial LN at 17 dpi. Scale bars for mandibular and tracheobronchiolar LNs: 100 µm. Scale bar for spleen: 500 µm. H&E, hematoxylin and eosin; IHC, immunohistochemistry.

CDV inoculation resulted in high-level infection of all lymphoid tissues and organs, which included not only the tissues and organs in close proximity to the respiratory tract, but also the more distant ones. In proximity to the respiratory tract, we observed macroscopic fluorescence in the tonsils, mandibular LN (Fig. 8A and B), tracheobronchial LN, and retropharyngeal LN. More distal, macroscopic fluorescence was detected in the thymus, spleen, Peyer’s patches (Fig. 8C through E), axillary LN, inguinal LN, and mesenteric LN.

**Fig 9:**
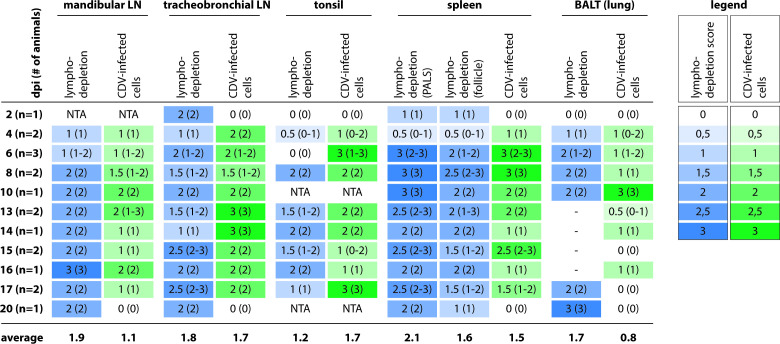
Semi-quantitative scoring of lymphodepletion and rCDV^RI^Venus(6)-infected cells in different lymphoid tissues. Lymphodepletion scores: 0: none; 1: mild; 2: moderate; 3: severe. Canine distemper virus (CDV)-infected cells scores: 0: none; 1: 1–10% CDV^+^ cells; 2: 11–40% CDV^+^ cells; 3: >40% CDV^+^ cells. Median scores with ranges were shown for detection of lymphodepletion and CDV-infected cells. Cells are color-coded from light (low score) to dark (high score). -, not present in sections; BALT, bronchus-associated lymphoid tissues; dpi, days post-inoculation; NTA, no tissue available; PALS, periarteriolar lymphoid sheaths.

IHC was performed to detect CDV-infected cells *in situ*. We performed a semi-quantitative scoring of CDV-infected cells in mandibular and tracheobronchial LNs, tonsils, and spleens isolated at different time points (Fig. 9). Infected cells were detected as early as 4 dpi in these tissues. In mandibular and tracheobronchial LNs, the percentages of infected cells reached the highest infection score (>40%) at 13 dpi or later, while in tonsils and spleens, the highest infection percentages were reached already at 6 dpi. To investigate whether CDV infection resulted in lymphocyte depletion in different lymphoid tissues over the course of infection, we scored the lymphodepletion in tonsils, mandibular and tracheobronchial LNs, spleens, and bronchus-associated lymphoid tissues in the lungs (Fig. 8F; Fig. 9). For most of the lymphoid tissues, severe lymphodepletion occurred around 16 dpi or later. In splenic periarteriolar lymphoid sheaths and follicles, severe lymphodepletion occurred earlier at 6 and 8 dpi, respectively, which coincided with the detection of higher infection percentages in spleens.

**Fig 10:**
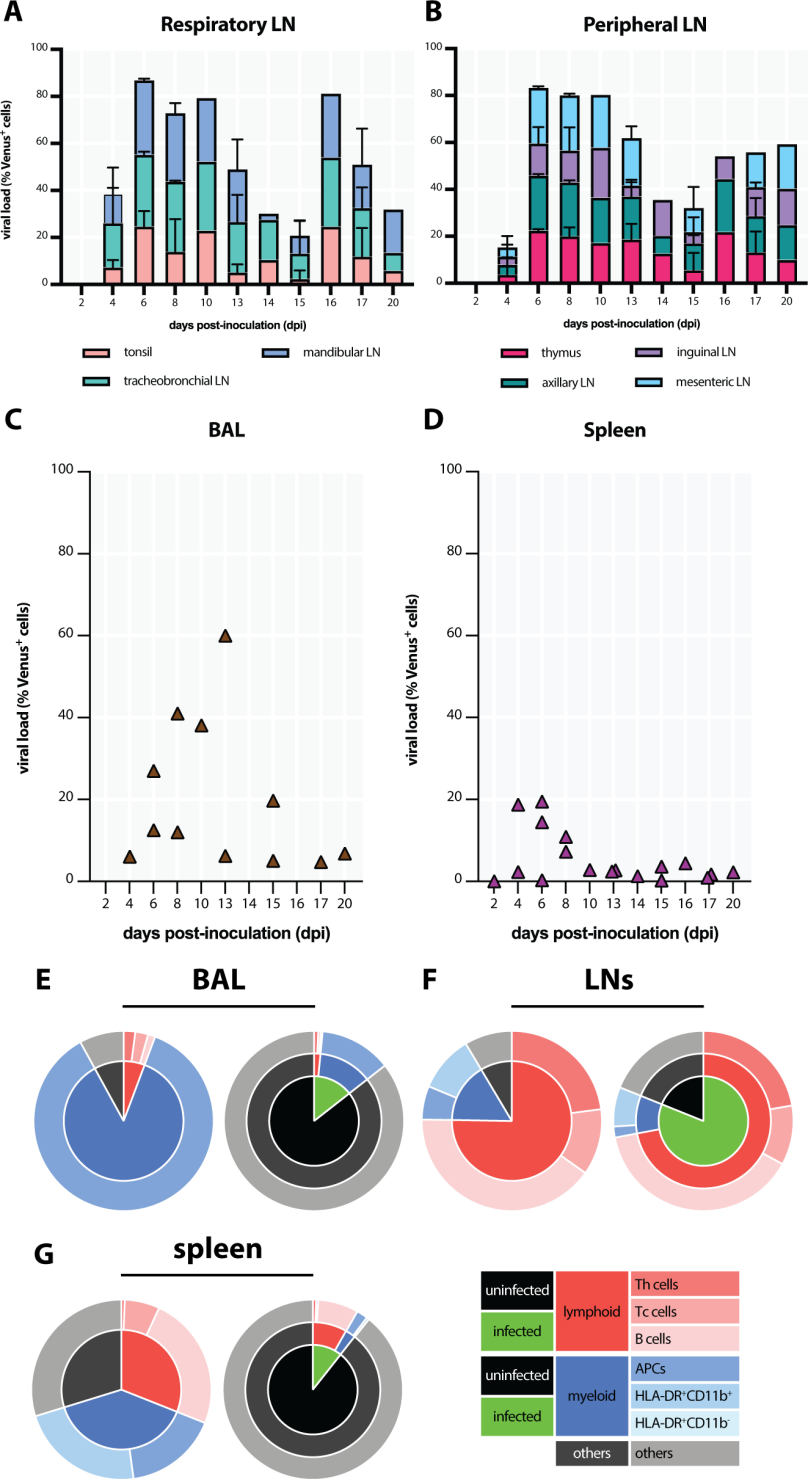
rCDV^RI^Venus(6) infects and depletes lymphoid cells. The percentages of rCDV^RI^Venus(6)-infected cells were determined in (A) respiratory lymph nodes (LNs) (tonsils, mandibular, and tracheobronchial LNs), (B) peripheral LNs (thymus, axillary, inguinal, and mesenteric LNs), (C) bronchoalveolar lavage (BAL), and (D) spleen obtained upon necropsy. Two animals that survived rCDV^RI^Venus(6) infection were not included in the analysis. (E–G) The distribution of different lymphoid and myeloid cells (left pie chart) and the phenotypes of infected cells (right pie chart) in (E) BAL, (F) lymph nodes, and (G) spleen at 6 days post-inoculation (dpi). At the peak of infection, canine distemper virus-infected cells in BAL comprised of myeloid cells, while in LNs and spleen, the majority of infected cells were lymphoid cells. Myeloid cells in BAL were not further subdivided and therefore were defined as antigen-presenting cells (APCs) in this figure.

We determined the viral loads in organs of rCDV^RI^Venus(6)-infected animals by generating single-cell suspensions and measuring the percentage of fluorescent cells by flow cytometry. Two animals that survived the rCDV^RI^Venus(6) infection were excluded from this analysis. Furthermore, several post-mortem broncho-alveolar lavages (BAL) were performed to determine the percentages of infected cells in the BAL. High percentages of CDV-infected cells were detected in respiratory (tonsils, mandibular, and tracheobronchial) and peripheral (thymus and axillary, inguinal, and mesenteric) lymphoid tissues as early as 4 dpi and remained detectable until the end of the study period (Fig. 10A and B). CDV-infected cells were also present in the BAL cells (Fig. 10C), but infection percentages were relatively low. Surprisingly, despite the detection of fluorescent splenic follicles (Fig. 8D), the percentages of CDV-infected WBC isolated from the spleen were rather low (Fig. 10D), compared to those in LNs. The percentages displayed in Fig. 10A through D were determined in tissues obtained at necropsy of animals euthanized at different time points, and should not be regarded repeated measures over time (Table 1).

Next, we characterized the cell composition and the phenotypes of CDV-infected cells present in BAL, LNs, and spleen by multicolor flow cytometry. In BAL, only a small fraction of lymphocytes could be detected, while the majority of the cells were of myeloid origin. As expected, the CDV-infected population in BAL comprised mostly of myeloid cells (Fig. 10E). In contrast, the single-cell suspensions obtained from LNs were dominated by lymphocytes, which were also the most prominent phenotype of CDV-infected cells, with a smaller fraction of CDV-infected myeloid cells (Fig. 10F). The spleen, unlike the BAL and the LNs, contained almost equal fractions of lymphocytes and myeloid cells. B cells were the predominant CDV-infected population in this organ (Fig. 10G).

**Fig 11:**
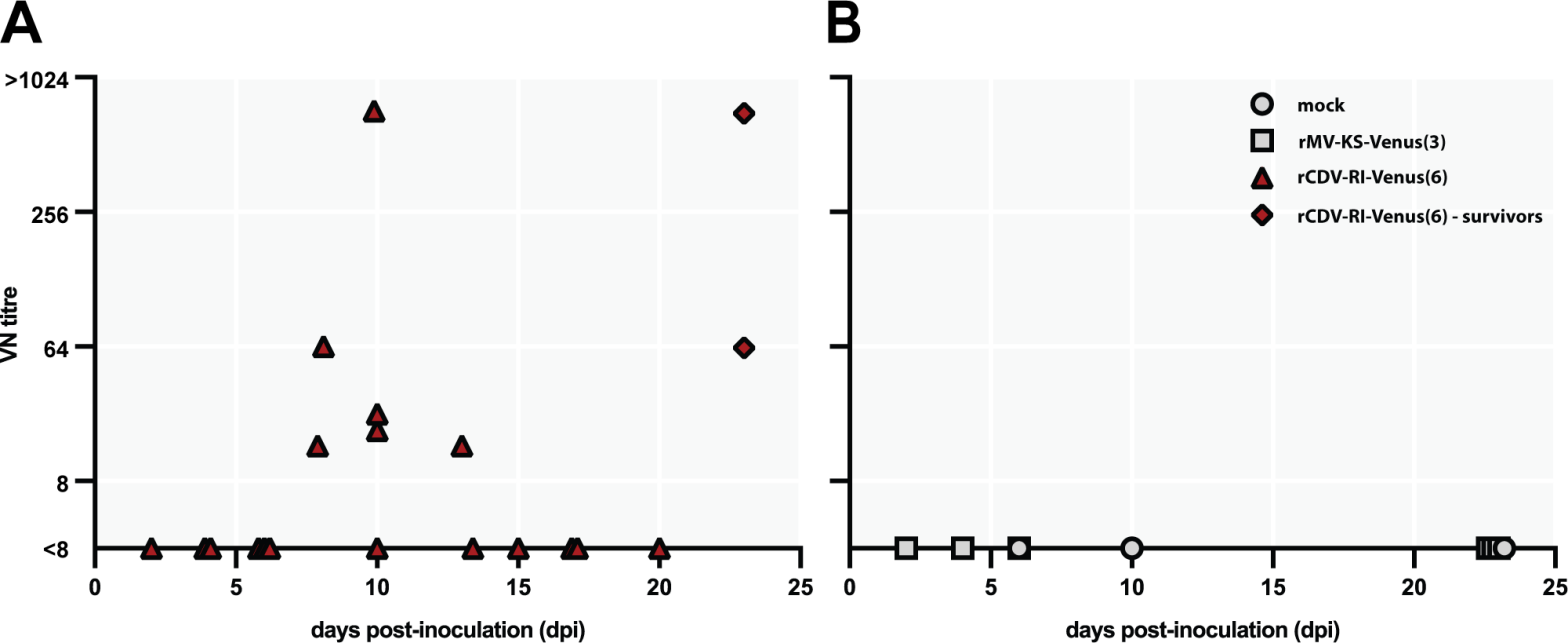
rCDV^RI^Venus(6) overwhelmed the humoral immune response in the majority of the animals. (A) The majority of animals (12 out of 20 animals) inoculated with rCDV^RI^Venus(6) did not seroconvert. Two animals that managed to clear the rCDV^RI^Venus(6) infection became seropositive at the end of the study period. (B) Mock-inoculated animals did not develop neutralizing antibodies. Each point represents individual animal and different shapes represents different groups. Square, rMV^KS^Venus(3)-infected animals; circle, uninfected animals.

Finally, we performed a VN test to detect the presence of CDV-specific neutralizing antibodies following infection. The majority of animals (12 out of 20 animals) inoculated with rCDV^RI^Venus(6) did not seroconvert at all (Fig. 11A and B). Six rCDV^RI^Venus(6)-infected animals developed low titers of neutralizing antibodies (<64), of which three were euthanized at early time points and another three were euthanized upon reaching humane endpoints. Both animals that survived the rCDV^RI^Venus(6) infection were positive for neutralizing antibodies. Altogether, this observation showed that CDV overwhelms the immune system, rarely leading to a functional humoral immune response.

## DISCUSSION

In this study, we show an overview of the pathogenesis of a novel wild type-based rCDV strain in ferrets. CDV replicated mainly in lymphoid tissues, but was also detected in the respiratory tract, at mucosal sites, in the skin, and in the CNS. The main target cells were myeloid and lymphoid cells, leading to cell-associated viremia and systemic dissemination. However, epithelial cell infection was also observed, especially in the respiratory tract and the skin. In some animals, the infection reached the CNS. CDV infection and depletion of lymphocytes resulted in severe lymphopenia. As a result, infected animals rarely produced detectable levels of CDV-specific neutralizing antibodies. While several aspects of our study overlap with previously published CDV pathogenesis studies, the main novelty of our study is the combination of clinical signs, classical virology, macroscopic imaging, detailed flow cytometry (including identification of CDV-infected immune cell subsets), and histopathological and immunohistochemical analysis within a single study.

Previous studies that used rCDVs have shown a different clinical presentation and disease progression *in vivo* (8, 10). rCDV^RI^ is based on an early passage virus isolate obtained from a naturally infected raccoon. The isolate was obtained by inoculating an oral swab of a free-ranging raccoon found dead in Rhode Island (USA, 2012) onto Vero cells expressing canine CD150 (Vero-dogCD150 cells). A first passage of the virus was used to obtain a viral consensus sequence (GenBank accession no. KU666057) from which the recombinant virus was constructed (12). We conclude that this strain is a good representative of contemporary wild-type CDV strains and thus suitable for use in distemper pathogenesis studies.

Whereas MV strains are mostly similar in biological properties and *in vivo* virulence, CDV strains tend to show more biological variation. This is not due to cell culture adaptation, but can be explained by other factors. In particular, MV naturally circulates in humans as a single host species, as we are the only primate species that lives in populations large enough to sustain endemic MV transmission. Despite being able to adapt to and infect diverse organs and cause systemic infection, the respiratory route remains the main transmission route for MV. In contrast, CDV infects many different carnivorous and omnivorous host species, and has the opportunity to adapt to each of these species (6). CDV is not only restricted to the respiratory mode of transmission, but can potentially be transmitted by grooming, fighting, biting, and consuming an infected animal species. Thus, compared to MV, CDV has more evolutionary barriers: not only within one host (inter-organ adaptation), but also from one host species to another (inter-host adaptation). These evolutionary barriers may thus result in a richer genetic diversity of CDV as compared to MV. It would be highly interesting to compare the biological properties of rCDV^RI^ with those of other CDV strains of known provenance and low *in vitro* passage history.

Studying the pathogenesis of measles often relies on clinical studies or experimental infection of NHPs, since the species is naturally susceptible to MV infection. The use of these animals in experiments raises ethical concerns. Rodents have been used as an alternative animal model to study the pathogenesis of measles, but are not naturally susceptible to wild-type MV infection and hence are not a good representation of the infection in humans (16, 17). MV has a similar tropism to its close relative CDV and the infection results in similar pathogenesis and disease presentation. Both infections also lead to the depletion of lymphoid cells. In measles, however, lymphopenia is transient and masked by rapid replenishment of new lymphocytes. The infection and depletion of memory lymphocytes causes immune amnesia and increased risks to secondary infections (15, 18 - 20). Nevertheless, measles patients usually develop strong MV-specific humoral and cellular immune responses, and the majority of measles patients recover without complications. This is different in canine distemper, where viral loads are much higher and persist longer, and the depletion of lymphocytes is much more substantial due to the higher infection percentages in both circulating and tissue-resident lymphocytes. Since CDV infection overwhelms the immune system and is often not cleared, this leads to severe morbidity, including secondary infections and neurological complications, and high mortality levels (21). The case-fatality rate of CDV infection depends on the infected species, but ranges from 50% in dogs to close to 100% in ferrets (22). Differences in disease outcome between MV and other morbilliviruses, like CDV, are possibly due to the replication kinetics. In our study, the peak of CDV viremia was be detected 6 d after infection, while MV replication is often still substantial in the second week after infection (15). We speculate that the rapid replication kinetics overwhelms the host before protective immunity is formed, resulting in severe disease. Indeed, when mathematically modelled, a higher morbillivirus polymerase activity leads to an infection that cannot be cleared (7). Despite these differences, CDV is an attractive model to study the pathogenesis of MV infection and measles-associated immune amnesia, especially in ferrets. Rational attenuation of CDV that results in a self-limiting infection in ferrets could thus provide an important tool to be able to model the course of MV infection in humans (23).

The tropism of animal morbilliviruses is not well studied and most insights come from MV tropism studies, in which the virus is shown to primarily infect lymphoid cells, most especially the memory cells, and myeloid cells, such as DCs and macrophages. The availability of cross-reactive antibodies for the identification of these immune cells in other species remains one of the limiting factors in the study of morbillivirus tropism. In our study, we showed for the first time that lymphoid and myeloid cells are the primary target cells of CDV and that the infection resulted in depletion of specific lymphocyte subsets.

Natural CDV and other animal morbillivirus infections often do not lead to induction of neutralizing humoral immune responses (24). CDV infection in dogs results in lymphopenia and immune suppression and those that survive the infection have robust humoral and cellular immune responses (25). Neutralizing antibodies can also be absent in seals infected with PDV (26). This lack of formation of virus-specific immunity often coincides with fatal disease. In our study, we did not observe the presence of neutralizing antibodies in the majority of animals inoculated with rCDV^RI^Venus(6). Six infected animals seroconverted within 2 wk, with one animal showing a high titer of neutralizing antibodies. Whether this animal would have been able to survive the infection remained a possibility, since the animal was sacrificed to study pathogenesis at an early time point. However, given that all animals were lymphopenic, the risks of secondary infections and developing other fatal diseases after recovering from distemper were still high.

CDV infection is commonly associated with neurological complications. In our model, we observed macroscopic fluorescence in the olfactory bulb of two of the animals infected with CDV, suggesting the importance of this tissue as the entry site for the virus to the brain. The importance of olfactory bulb as one of the pathways used by morbilliviruses, such as CDV, to access the CNS has been highlighted in previous studies (27). However, no neurological changes nor spread throughout the brain were observed in this study. We speculate that the absence of observable neurological changes was due to the restricted duration of the study. The animals were kept for maximum 23 d after inoculation, but often sacrificed earlier, while neurological changes might only become apparent at later time points. We also observed the presence of CDV-infected cells in the choroid plexus and meninges. This observation was in accordance to previous studies that show that CDV can use multiple pathways to access the CNS, including through circulating CDV-infected lymphocytes in the choroid plexus (27).

In summary, we showed how inoculation of ferrets with a rCDV based on a wild-type strain resulted in widespread infection of myeloid, lymphoid and epithelial cells. This virus is a promising tool to further study the pathogenesis of CDV infections. Moreover, studies of CDV pathogenesis, not only focusing on the similarities but also on the differences between CDV and MV, will contribute to our understanding of the group of the morbilliviruses at large, and of MV as an important human pathogen.
